# Microbial contamination of the hands of healthcare providers in the operating theatre of a central hospital

**DOI:** 10.4102/sajid.v36i1.221

**Published:** 2021-04-08

**Authors:** Kylesh D. Pegu, Helen Perrie, Juan Scribante, Maria Fourtounas

**Affiliations:** 1 Department of Anaesthesiology, Faculty of Health Sciences, University of the Witwatersrand, Johannesburg, South Africa

**Keywords:** hands, healthcare providers, commensal, pathogen, microorganism

## Abstract

**Background:**

Effort is invested in maintaining the sterility of the operating field, but less attention is paid to potential healthcare associated infection (HAI) sources through patient contact with non-scrubbed healthcare providers (HCPs). A single microbiological assessment of hands can provide a good assessment of the potential dynamic transmission of microorganisms. The aim of this study was to identify and quantify the microbial growth on the hands of HCPs in the operating theatres of Chris Hani Baragwanath Academic Hospital.

**Methods:**

A prospective, contextual and descriptive study design was followed. Seventy-five samples were collected using convenience sampling from an equal number of surgeons, anaesthetists and nurses. Specimens were taken using agar plates and underwent semi-quantitative analysis.

**Results:**

All the hands of the HCPs displayed growth; 95% grew commensals and 64% grew pathogens. Eighteen commensal microorganisms and 21 pathological microorganisms were noted. Comparisons of commensal, pathological and combined levels of contamination among the three groups were not statistically significant (*p* = 0.061, *p* = 0.481, *p* = 0.236). No significant difference between the growth of combined microorganisms (*p* = 0.634) and pathological microorganisms (*p* = 0.499) among the groups. Surgeons had significantly more commensal growth (*p* = 0.041). There was no statistically significant difference between sexes (*p* = 0.290).

**Conclusion:**

It was concerning that 100% of the hands of HCPs who were about to commence with the surgical list had microbial growth. These HCPs could have already been in contact with patients and equipment in the theatre environment.

## Introduction

Healthcare-associated infections (HAIs) are infections that appear in a patient under medical care that were not present at the time of admission.^1^ The incidence of HAIs in developed countries ranges from 3.5% to 12%, whilst in developing countries it ranges from 5.7% to 19.1%.^2^ Patients who develop an HAI remain in hospital two and a half times longer, with hospital costs nearly three times higher, and incur further medical costs after discharge from hospital when compared to uninfected patients.^3^ The most frequent types of HAI include central line-associated bloodstream infections, catheter-associated urinary tract infections, surgical site infections and ventilator-associated pneumonia.^1^ The risk factors for developing an HAI include poor hygienic conditions of the healthcare setting, increased patient susceptibility, inadequate hand hygiene and poor knowledge of infection control policies.^4^

In the operating theatre (OT), much effort is invested in maintaining the sterility of the operating field, but less attention is paid to potential HAI sources resulting from patient contact with non-scrubbed healthcare providers (HCPs).^5^ The microorganisms present on the hands of HCPs serve as a reservoir for potential contamination. In the OT, contamination of the hands of HCPs can independently increase the risk of patients being contaminated.^6^

Loftus et al.^7^ conducted a study where the phenotypes of *Staphylococcus aureus* isolated from HCPs’ hands were linked phenotypically to patients’ 30-day post-operative cultures. Pathogenic microorganisms cultured from the hands of HCPs include coagulase-negative *Staphylococci, Acinetobacter baumannii, Klebsiella pneumoniae, Proteus mirabilis, Enterobacter cloacae, Staphylococcus aureus, Pseudomonas aeruginosa, Escherichia coli* and methicillin-resistant *Staphylococcus aureus.*^8^ The intraoperative environment serves as a risk factor for the development of HAIs.^5^ The failure to conduct hand hygiene measures before and after patient contact can lead to contamination of the patient OT equipment, hence creating a reservoir for pathogens that can cross-infect the next patient.^5^ Non-compliance with hand hygiene practices by the non-scrubbed staff has increased microbial transmission to patients in the OT environment.^9^ Contamination of OT equipment such as telephones, keyboards, anaesthesia machines and stopcocks by hands with pathogens has been well documented.^5^ Unhygienic staff perform invasive procedures such as tracheal intubation and insertion of intravascular devices and urinary catheters, which enables pathogens to bypass the normal patient defence barriers and cause HAIs.^5^ The infective dose for many pathogens appears to be very low, and slight contamination of the environment is sufficient to cause infection.^10^

Ineffective hand hygiene is being practised globally.^1,2,3,4,5,11^ The duration of microorganism survival on hands differs with various microorganisms, with some able to survive for more than an hour.^10^ Inadequate hand hygiene often leads to the survival of these microorganisms causing increased risk of cross-transmission.^10^ Hand contamination has increased sequelae beyond the intraoperative risk of transmission.^12^ Directly observed behaviour of hand hygiene has shown low compliance to institutionally developed protocols.^5,13^

A single microbiological assessment of hands can provide a good assessment of the potential transmission.^14^ Limited South African studies reporting hand contamination of HCPs have been identified, either nationally or at Chris Hani Baragwanath Academic Hospital (CHBAH). The aim of this study was to identify and quantify the microbial growth on the hands of HCPs in the OTs of CHBAH.

## Methods

A prospective, contextual and descriptive study design was followed. Approval to conduct the study was obtained from the Human Research Ethics Committee (Medical) and other relevant authorities.

The study population consisted of HCPs (surgeons, anaesthetists and nurses) in the OTs of CHBAH. Convenience sampling was used, and because of financial constraints, 75 samples were collected, which allowed an equal number of participants in each of the three groups. After consultation with a biostatistician, a sample size of 75 participants at an expected contamination rate of 80% in all HCPs, with an average of 65% contamination found in the literature,^15,16,17,18,19,20^ will give a power of 84% at a significance level of 5%. All HCPs who consented to participate in the study were included and were only enrolled once.

In consultation with a microbiologist, it was decided to use a low-budget, high-volume process. Agar plates were used to collect specimens. This form of semi-quantitative analysis is cheap and requires minimal microbiological analysis and logistical support.^21^ Samples were collected on single days over 1 month to prevent HCPs from changing their practices of hand washing. The study was explained to the HCPs, and signed consent was obtained prior to specimen collection. Agar plating was conducted in the morning prior to the commencement of the surgical list. The collection process consisted of HCPs pressing the fingertips of their dominant hand, followed by the base of the same hand, into the agar plate for 5 s each.

The samples were collected by one author (K.P.), and a standard laboratory request form was used to enter each specimen’s differentiating information. The samples collected were stored at room temperature and were delivered to the laboratory at the earliest possible time on the same day. The samples were processed by an accredited laboratory, Vermaak and Partners Pathologists.

The samples were incubated for 48 h, after which the colonies were examined, tallied and detailed. For this study, a standard semi-quantitative criterion was used with assigned scores given to microorganisms:

- 1+ = rare
- 2+ = few
- 3+ = moderate
- 4+ = many

A distinction between pathological and commensal microorganisms was made; however, this was difficult as some commensal microorganisms can result in a nosocomial infection, and some pathological microorganisms can also be commensals in the appropriate setting. Participants could ask for their microbial results, and if a participant grew pathological organisms, they were informed of the growth.

The data were analysed with Stata version 16 (StataCorp USA). The categorical variables were described using frequencies and percentages. Comparisons between the levels of contamination among the groups were done using Fisher’s exact test. The Kruskal–Wallis test was used to compare the number of microorganisms among the three groups. The Dunn test was applied to any significant difference. A *p*-value of < 0.05 was considered statistically significant.

## Results

Seventy-seven HCPs were approached, but two declined. Samples were collected from 75 participants, which included 25 anaesthetists, 25 surgeons and 25 nurses. Of the participants, 69.3% were female and 30.7% were male. Eighteen commensal microorganisms and 21 pathological microorganisms were grown. All hands of the HCPs displayed growth, of which 95% cultured commensals and 64% cultured pathogens. No 4+ growth was noted. A table identifying and quantifying the microorganisms grown is included as supplementary data. Microorganisms were classified with the use of recent literature and with the assistance of a medical microbiologist.

The growth according to sex is shown in Table 1. A *p*-value of 0.290 was calculated, which was not statistically significant, for commensal, pathological and combined growth between the two sexes.

**TABLE 1:** Growth according to sex.

Professional designation	Total	Male	Female	Growth					
				Commensal		Pathological		Combined	
				Male	Female	Male	Female	Male	Female
Anaesthetist	25	8	17	3	6	0	0	5	11
Nurse	25	0	25	0	8	0	3	0	14
Surgeon	25	15	10	6	6	1	0	8	4

**Total**	**75**	**23**	**52**	**9**	**20**	**1**	**3**	**13**	**29**

The number of microorganisms cultured on the hands of HCPs is shown in Table 2. Seventy-six per cent of HCPs had two or more microorganisms on their hands.

**TABLE 2:** Number of organisms on healthcare provider hands.

Number of organisms	Number of HCPs	HCPs (%)
1	18	24.0
2	30	40.0
3	20	26.7
4	3	4.0
5	2	2.7
6	2	2.7

HCPs, healthcare providers.

**FIGURE 1:**
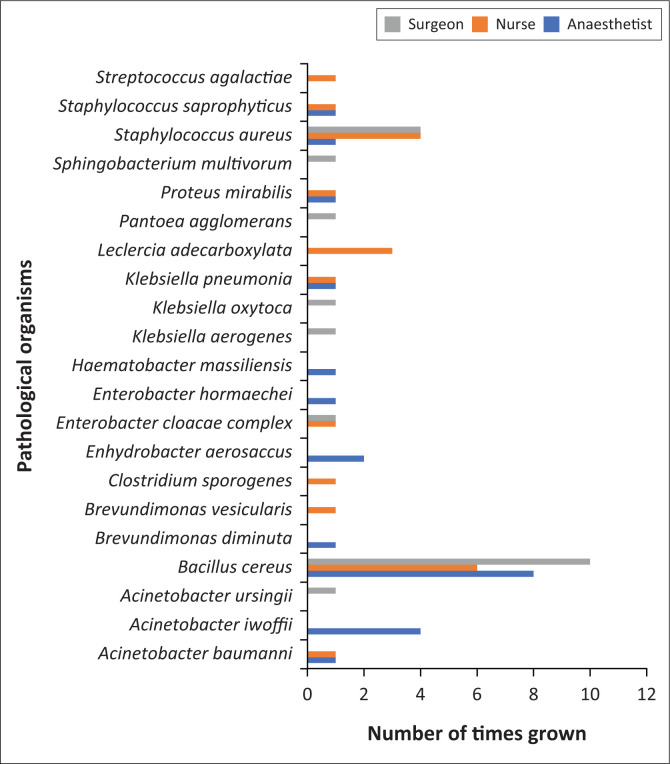
Pathological growth amongst each group of healthcare workers.

Figure 1 shows the pathological microorganisms and the number of times each was grown by each type of HCP.

**FIGURE 2:**
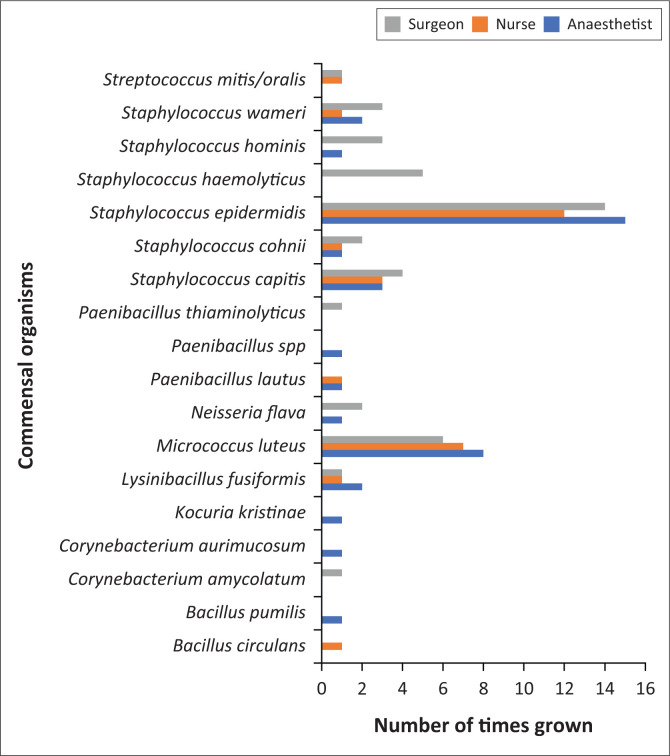
Commensal growth among each group.

Figure 2 shows the commensal microorganisms and the number of times they were grown amongst each group of HCPs.

The levels of contamination of microorganisms on the hands between the three groups of HCPs were compared. There were no significant differences found when combined (*p* = 0.061), commensal (*p* = 0.481) and pathological (*p* = 0.236) microorganisms were compared.

The number of microorganisms on the hands of HCPs in each group were compared. There was a significant difference in the growth of commensals between the groups (*p* = 0.041). A significant difference was present between nurses and surgeons (*p* = 0.009) and anaesthetists and surgeons (*p* = 0.052). The surgeons had the highest number of commensal microorganisms on their hands. There was no significant difference between the groups in the growth of pathological microorganisms (*p* = 0.499) and combined microorganisms (*p* = 0.634).

## Discussion

This study emphasises a significant level of microorganism contamination on the hands of HCPs; all the hands of the participants were contaminated, with 76% of the hands growing two or more microorganisms. A study in Wales by Al-Allak et al.^22^ found that 100% of HCPs’ hands were contaminated. Hand contamination ranged from 62.3% to 100% in studies conducted in various hospital settings in developed and developing countries.^17,20,23,24,25,26,27,28^ It is of concern that in our study, all participants were about to commence with the surgical list for that day and had already been in contact with the OT environment.

Commensal microorganisms were cultured in 95% of participants. Eighteen commensal microorganisms were cultured, with the most predominant being *Staphylococcus epidermis* (54.7%), *Bacillus cereus* (32%), *Micrococcus luteus* (28%) and *Staphylococcus capitis* (13.3%). *Staphylococcus epidermidis* may promote sepsis by its ability to form biofilms on indwelling medical devices and produce toxins.^29^ *Bacillus cereus* can cause localised infection and bacteraemia and can be associated with haematogenous spread.^30^ In haematological patients *Bacillus cereus* has the ability to invade the central nervous system.^30^ *Micrococcus luteus* has been implicated in HAIs in immunocompromised patients.^31^ *Staphylococcus capitis* can be considered a pathogen in neonates, and a drug-resistant form has emerged as a cause of sepsis in neonatal intensive care units.^32^ The microorganisms grown in our study are in keeping with commensal strains.^33^ Commensal microorganisms may cause infection if they enter a sterile body cavity,^34^ may permanently colonise the hands of HCPs and are often associated with HAIs.^35^

Pathological microorganisms were cultured in 64% of participants. Twenty-one pathological organisms were grown, with the most predominant being *Staphylococcus aureus* (12%), *Acinetobacter iwoffii* (5.3%) and *Leclercia adecarboxylata* (4%). *Staphylococcus aureus* has the propensity to develop resistance to antimicrobial agents and is one of the most lethal bloodstream pathogens.^36^ *Acinetobacter iwoffii* can cause HAIs in patients with chronic illnesses andincreases the length of hospitalisation and mortality.^37^ *Leclercia adecarboxylata* commonly affects immunocompromised individuals, and whilst being susceptible to antibiotics, resistant strains have now been identified.^38^

Thirty-nine microorganisms were cultured in total. The microorganism count was more than in other studies; Rocha et al.^39^ grew 11 microorganisms, Wong et al.^17^ grew 20 microorganisms, Sureshkumar et al.^27^ grew 12 microorganisms and Larson^40^ grew 14 microorganisms. Possible reasons for microorganism growth could be ineffective hand hygiene, damaged skin and the local healthcare microbial environment.^1,39,40,41^

There was no significant difference between the level of growth of microorganisms on the hands of anaesthetists, nurses and surgeons. No differences were noted between professional designations in prior studies, indicating that this does not influence the microbial environment of hands.^16,20,42^ When comparing the number of microorganisms present on the hands of HCPs, there was no significant difference for combined and pathological growth. The surgeons’ hands had a higher number of commensal microorganisms present when compared to anaesthetists and nurses. The surgeons are possibly exposed to a different microbial environment by performing work in wards prior to arriving in theatre.

Of the studies assessing the hand hygiene of anaesthetists, only two gave total growth values that ranged from 66% to 71%.^43,44^ Anaesthetists in this study had a 100% growth rate from their hands whilst growing more microorganisms. The 100% growth rate from the hands of nurses was higher than the growth rates identified in two studies, ranging from 5.1% to 77.5%.^40,45^ The growth from this study was in keeping with studies assessing damaged skin and poor nail hygiene.^40,45^ There were no specific studies identified that assessed the hand hygiene of only surgeons in the OT environment.

The population was skewed with regard to sex, as 69.3% females and 30.7% males participated in the study. However, there was no statistically significant difference between the sexes. No study was identified that showed differences with microorganisms between males and females. The analysis was done because of females possibly having longer nails, nail polish and more rings when compared to males. This was not further analysed as this was not an objective of the study.

The results from this study indicate the need for adequate hand hygiene practice. A simple hand hygiene model, such as ‘My Five Moments of Hand Hygiene’, has had low compliance in healthcare settings.^46,47^

Altering poor hand hygiene practice requires a multidimensional model targeting knowledge, attitude and clinical skills.^48^ Elective hand hygiene is difficult to correct and will be challenging to adjust to an implemented guideline.^49^ Barriers to hand hygiene, which are similar in our healthcare setting, are high workloads, too few personnel, unfavourable hand hygiene materials and structures and high infection risk activities.^48^ Limitations of this study included a contextual analysis of the microorganisms present. The funding of the study was a limitation because a semi-quantitative analysis was done; however, a single microbiological assessment of hands can provide a good assessment of the potential transmission. The pandemic caused by severe acute respiratory syndrome coronavirus 2 (SARS-CoV-2) has emphasised the need for compliance with hand hygiene measures.

## Conclusion

It was concerning that 100% of the hands of HCPs who were about to commence with the surgical list had microbial growth. These HCPs could have already been in contact with patients and equipment in the theatre environment. Microorganisms cultured on hands are a source of cross-transmission, which may result in HAIs. Institutions require the implementation of a multidimensional model to amend and implement guidelines and to increase the awareness and availability of hand hygiene materials.

## Appendix 1

**TABLE 1-A1:** Identity and quantity of organisms grown.

Organism	No of times grown (%)	Level of growth		
		1+	2+	3+
**Commensal organisms**				
*Bacillus cereus*	24 (32)	16	7	1
*Bacillus pumilis*	1 (1.3)	1	0	0
*Corynebacterium amycolatum*	1 (1.3)	1	0	0
*Corynebacterium aurimucosum*	1 (1.3)	0	1	0
*Kocuria kristinae*	1 (1.3)	1	0	0
*Lysinibacillus fusiformis*	4 (5.3)	3	1	0
*Micrococcus luteus*	21(28)	6	12	3
*Neisseria flava*	3 (4.0)	2	1	0
*Paenibacillus lautus*	2 (2.7)	2	0	0
*Paenibacillus spp*	1 (1.3)	1	0	0
*Paenibacillus thiaminolyticus*	1 (1.3)	1	0	0
*Staphylococcus capitis*	10 (13.3)	5	4	1
*Staphylococcus cohnii*	4 (5.3)	1	2	1
*Staphylococcus epidermidis*	41 (54.7)	22	17	2
*Staphylococcus haemolyticus*	5 (6.6)	1	3	1
*Staphylococcus hominis*	4 (5.3)	2	2	0
*Staphylococcus wameri*	6 (8.0)	5	1	0
*Streptococcus mitis/oralis*	2 (2.7)	1	1	0
**Pathological organisms**				
*Acinetobacter baumanni*	2 (2.7)	1	1	0
*Acinetobacter iwoffii*	4 (5.3)	4	0	0
*Acinetobacter ursingii*	1 (1.3)	0	1	0
*Bacillus circulans*	1 (1.3)	1	0	0
*Brevundimonas diminuta*	1 (1.3)	0	1	0
*Brevundimonas vesicularis*	1 (1.3)	0	1	0
*Clostridium sporogenes*	1 (1.3)	1	0	0
*Enhydrobacter aerosaccus*	2 (2.7)	2	0	0
*Enterobacter cloacae complex*	2 (2.7)	0	2	0
*Enterobacter hormaechei*	1 (1.3)	0	1	0
*Haematobacter massiliensis*	1 (1.3)	0	1	0
*Klebsiella aerogenes*	1 (1.3)	1	0	0
*Klebsiella oxytoca*	1 (1.3)	0	1	0
*Klebsiella pneumonia*	2 (2.7)	0	2	0
*Leclercia adecarboxylata*	3 (4.0)	0	1	2
*Pantoea agglomerans*	1 (1.3)	0	1	0
*Proteus mirabilis*	2 (2.7)	1	1	0
*Sphingobacterium multivorum*	1 (1.3)	0	1	0
*Staphylococcus aureus*	9 (12)	5	3	1
*Staphylococcus saprophyticus*	2 (2.7)	0	1	1
*Streptococcus agalactiae*	1 (1.3)	1	0	0
